# Generation and Characterization of Live Attenuated Influenza A(H7N9) Candidate Vaccine Virus Based on Russian Donor of Attenuation

**DOI:** 10.1371/journal.pone.0138951

**Published:** 2015-09-25

**Authors:** Svetlana Shcherbik, Nicholas Pearce, Amanda Balish, Joyce Jones, Sharmi Thor, Charles Todd Davis, Melissa Pearce, Terrence Tumpey, David Cureton, Li-Mei Chen, Julie Villanueva, Tatiana L. Bousse

**Affiliations:** 1 Influenza Division, National Center for Immunization and Respiratory Diseases, Centers for Disease Control and Prevention, Atlanta, GA, United States of America; 2 Battelle, Atlanta, GA, United States of America; 3 Atlanta Research and Education Foundation, Atlanta, GA, United States of America; Thomas Jefferson University, UNITED STATES

## Abstract

**Background:**

Avian influenza A (H7N9) virus has emerged recently and continues to cause severe disease with a high mortality rate in humans prompting the development of candidate vaccine viruses. Live attenuated influenza vaccines (LAIV) are 6:2 reassortant viruses containing the HA and NA gene segments from wild type influenza viruses to induce protective immune responses and the six internal genes from Master Donor Viruses (MDV) to provide temperature sensitive, cold-adapted and attenuated phenotypes.

**Methodology/Principal Findings:**

LAIV candidate A/Anhui/1/2013(H7N9)-CDC-LV7A (abbreviated as CDC-LV7A), based on the Russian MDV, A/Leningrad/134/17/57 (H2N2), was generated by classical reassortment in eggs and retained MDV temperature-sensitive and cold-adapted phenotypes. CDC-LV7A had two amino acid substitutions N123D and N149D (H7 numbering) in HA and one substitution T10I in NA. To evaluate the role of these mutations on the replication capacity of the reassortants in eggs, the recombinant viruses A(H7N9)RG-LV1 and A(H7N9)RG-LV2 were generated by reverse genetics. These changes did not alter virus antigenicity as ferret antiserum to CDC-LV7A vaccine candidate inhibited hemagglutination by homologous A(H7N9) virus efficiently. Safety studies in ferrets confirmed that CDC-LV7A was attenuated compared to wild-type A/Anhui/1/2013. In addition, the genetic stability of this vaccine candidate was examined in eggs and ferrets by monitoring sequence changes acquired during virus replication in the two host models. No changes in the viral genome were detected after five passages in eggs. However, after ten passages additional mutations were detected in the HA gene. The vaccine candidate was shown to be stable in the ferret model; post-vaccination sequence data analysis showed no changes in viruses collected in nasal washes present at day 5 or day 7.

**Conclusions/Significance:**

Our data indicate that the A/Anhui/1/2013(H7N9)-CDC-LV7A reassortant virus is a safe and genetically stable candidate vaccine virus that is now available for distribution by WHO to vaccine manufacturers.

## Introduction

The emergence of avian influenza A (H7N9) viruses in China with a high mortality rate in humans poses a significant global health concern [1–3]. While mild illnesses in human H7N9 patients have been observed, most patients have had severe respiratory illness, with about one-third of cases resulting in death. As of February 23 2015, five hundred seventy-one laboratory-confirmed cases of human infection with avian influenza A(H7N9) virus, including two hundred twelve deaths, have been reported to WHO since February 2013 (http://www.who.int/influenza/human_animal_interface/influenza_h7n9/RiskAssessment_H7N9_23Feb20115.pdf). Vaccines are still the best countermeasure against emerging influenza virus infections with pandemic potential. Antigenic analysis of influenza A(H7N9) viruses isolated indicates that the hemagglutinin (HA) and neuraminidase (NA) genes of the viruses remain similar, and all viruses are antigenically close to A/Anhui/1/2013 (H7N9) virus which was recommended for vaccine development by the WHO (http://www.who.int/influenza/vaccines/virus/candidates_reagents/summary_a_h7n9_cvv_20150317.pdf?ua=1).

Live attenuated influenza vaccine (LAIV) are based on cold-adapted, temperature-sensitive vaccine virus strains that replicate in the nasopharynx but poorly in the lower respiratory tract. LAIVs are 6:2 reassortant viruses containing the HA and NA gene segments from wild type influenza viruses to induce protective immune responses and the six internal gene segments from a Master Donor Viruses (MDV) to provide temperature sensitive, cold-adapted and attenuated phenotypes. Two types of LAIVs are available commercially. The first, licensed as FluMist (MedImmune, Inc.), is based on A/Ann Arbor/6/60 influenza A and B/Ann Arbor/1/66 influenza B; it is currently produced using seed viruses made by reverse genetics [4]. LAIVs based on the Russian MDV strains, A/Leningrad/134/17/57 (H2N2) and B/USSR/60/69, are made using seed viruses produced by conventional reassortment in eggs and have been used safely for more than 50 years in Russia [5–9]. Through cooperation with the WHO, production and use of seasonal LAIV vaccine based on Russian donors of attenuation was expanded internationally to India, Thailand and China [10–12]. The increased international demand of Russian LAIV reassortant viruses prompted the WHO to establish an additional facility at the Centers of Disease Control and Prevention (CDC), Influenza Division to prepare and incorporate quality assessment of LAIV reassortants for international use.

Several studies have shown that A(H7N9) influenza virus appears to be capable of evading human cellular and humoral immune response [13–16]. There was a relatively weak protective antibody response detected in serum of patients infected with influenza A(H7N9) in Shanghai and Beijing, China [13]. H7 HA containing viruses and vaccines have been shown to be poorly immunogenic in general [13–16]. The poor immunogenicity of H7 viruses was attributed to the H7 hemagglutinin properties [17]. Several A/Anhui/1/2013(H7N9)-like inactivated vaccine types were developed and tested in animal protection studies–whole virus vaccine [18], split vaccine [15, 19, 20], recombinant virus-like particles [15, 21], cell-based whole virus vaccine [22, 23]. In all studies either several doses of administration or various adjuvants were required for complete protection. LAIV A/Anhui/1/2013 (H7N9) seed virus developed on A/AnnArbor/6/60 backbone [24] however, demonstrated the complete protection of ferrets from homologous wild-type (*wt)* A/Anhui/1/2013 (H7N9) virus after a single dose, another study showed protective efficacy of similar LAIV virus in a mouse model [25]. The clinical studies of A(H7N9) vaccine suggest that to achieve protective immunity against subtype A(H7N9) viruses multiple vaccinations might be required [26–28]. A recent study found that the quantity, epitope diversity, and affinity of H7 head-specific antibodies increased rapidly after the vaccination with inactivated vaccine in LAIV-primed subjects only, emphasizing the value of LAIVs as a tool for pre-pandemic vaccination [29]. The development of LAIV which could be used in countries with pandemic potential is of high importance. The preferable method approved by WHO for LAIV preparation is reassortment in eggs, in the case of newly emergent, potentially pandemic viruses, a reverse genetics approach is also accepted [30, 31]. However, due to intellectual property issues currently present for reverse genetics generated LAIV vaccines, the production of such vaccines could be costly, which is a concern for developing countries manufacturers (http://www.who.int/phi/Day1_Session3_PATH_Marks.pdf).

In this study, we report the generation of a live attenuated influenza vaccine against A/Anhui/1/2013 (H7N9) virus using classical reassortment The vaccine candidate contained the HA and NA genes derived from A/Anhui/1/2013(H7N9) and six internal genes of MDV A/Leningrad/134/17/57 virus. The vaccine candidate generated by classical reassortment in embryonated chicken eggs acquired two amino acid substitutions in HA (N123D and N149D, H7 numbering) and one in NA T10I. To evaluate the effect of these mutations on the replication capacity of the reassortants in eggs, two recombinant viruses were generated by reverse genetics—A(H7N9)RG-LV1, which contained the HA and NA identical to original *wt* A/Anhui/1/2013, and A(H7N9)RG-LV2, which had mutations in HA and NA similar to CDC-LV7A. The egg selected mutations in candidate vaccine virus were also evaluated for their effect on antigenicity. The final live vaccine candidate was evaluated for its homogeneity, genetic stability and safety in the ferret model.

## Materials and Methods

### Viruses and cells

Egg adapted *wt* influenza virus strain A/Anhui/1/2013 (H7N9) and other A(H7N9) viruses, A/Netherlands/219/2003, A/Mallard/ Netherlands /12/2000, A/Shanghai/1/2013, A/Shanghai/2/2013, A/Taiwan/1/2013, A/Hong Kong/5942/2013, A/Jiangsu/1/13, A/Zhejiang/2/2013, A/Shanghai/7/2013, A/Hong Kong/ 2212982/2014, A/Hong Kong/734/2014, A/Hong Kong/56/2015, A/Hong Kong/2550/2015, A/British Columbia/1/2015 were obtained from the CDC repository. Passage history E2/E1, where E#/ means number of egg passages in submission laboratory and /E# means number of egg passages at Center for Disease Control (CDC), Atlanta, GA. A/Leningrad/134/17/57 (H2N2) Master Donor Virus (MDV) was provided by Institute of Experimental Medicine (IEM), Russia/ BioDiem, Australia. The viruses used for vaccine candidate generation were propagated in 9 to 12-day-old specific pathogen free (SPF) eggs (Charles River Laboratories Inc., Wilmington, MA). All reassortment experiments between influenza A (H7N9) virus and MDV were performed in an approved biosafety level 3 (BSL3) containment laboratory. 293T human embryonic kidney (HEK) cells were obtained from American Type Culture Collection (ATCC) and were maintained in DMEM High Glucose (Life Technologies, Carlsbad, CA) supplemented with 10% fetal bovine serum (Life Technologies), 1x GlutaMAX (Life Technologies) and 40 μg/ml Neomycin (Sigma-Aldrich, St. Louis, MO).

### Classical reassortment in eggs

A reassortant influenza virus that possesses the internal genes of MDV and the surface antigen genes of *wt* A/Anhui/1/2013 was prepared according to the method developed by IEM, St. Petersburg, Russia [7, 32, 33] and optimized at CDC, Atlanta, USA. Briefly, donor and *wt* virus were inoculated into 10-day-old SPF eggs and incubated at 32°C for 2 days. HA-positive allantoic fluids (AF) were combined and diluted 1:10 using antiserum prepared against the MDV in ferrets. The virus-serum mixtures were incubated overnight at 4°C and then passaged once in SPF eggs at 25°C for 6 days, following the blind passage at 32°C. HA-positive AF were analyzed by HI assay for antigenic specificity with antiserum to MDV and *wt* influenza virus. AF which exhibited antigenic specificity of *wt* virus were combined and a cloning by limiting dilution procedure was carried out in the presence of antiserum at 25°C. 6:2 reassortants were selected as described previously [34].

### Isolation of viral RNA

RNA was isolated from influenza virus-containing allantoic fluids and purified on the MagnaPure LC (Roche, San Francisco, CA) using the MagNA Pure Total Nucleic Acid Kit (Roche) following the manufacturer’s instructions. Clarified allantoic fluid of infected eggs (200 μL) was used for RNA isolation. RNA was eluted in a final volume of 50 μL of water.

### Genotyping of reassortants

Genomic composition of the reassortant influenza viruses was assessed by pyrosequencing analysis of NA and the 6 internal genes. Pyrosequencing analysis was performed using the PyroMark Q96 ID Platform (Qiagen, Carlsbad, CA, USA) following the manufacturer’s instructions. RT-PCR with biotinylated primers, gel analysis of the RT-PCR product, sample preparation, pyrosequencing reaction, and data analysis were done as described previously [34]. The primers were designed based on sequences of the A/Anhui/01/2013 and MDV using PSQ Assay Design software (Qiagen).

### Real time RT-PCR

The genetic homogeneity of the final seed virus was confirmed by real time RT-PCR test as described previously [35]. Strain and segment specific primer/probe sets were designed for *wt* A/Anhui/1/2013 (H7N9) virus using PrimerExpress 3.0 Software (Applied Biosystems, Foster City, CA), the sequences are listed in S1 Table. The primer/probes sets used for MDV are reported elsewhere [35]. The rRT-PCR was performed using SuperScript III PlatinumOne-Step quantitative RT-PCR Kit (Life Technologies, Carlsbad, CA) on CFX96 Touch Real-Time PCR Detection System (Bio-Rad, Hercules, CA). Reactions were conducted in a total volume of 25 μl containing 0.8 μM of each primer and 0.2 μM of probe and 5 µl of viral RNA. Reaction conditions were as follows: one cycle of 50°C for 15 min, followed by 2 min at 95°C, and 40 cycles of 15 sec at 95°C and 1 min at 60°C. The data was analyzed using Bio-Rad CFX Manager 2.1 software.

Specificity and sensitivity of the real time RT-PCR assays were evaluated relative to the value of 50% egg infected dose (EID_50_). For sensitivity assay RNA was extracted from AF with a known viral titer, in EID_50,_ and serial 10-fold dilutions of extracted RNA were used for amplification with designed primer/probe sets. The sensitivity of the assays was determined as the lowest concentration at which positive signal was obtained.

### Hemagglutination inhibition (HI) assay

The HI assay was used to determine the origin of HA genes of reassortant virus clones. HI assays were performed in 96-well V-microtiter plates using 0.5% turkey red blood cells (tRBC) and antiserum against MDV or *wt* influenza viruses [36, 37].

### Phenotypic and genetic analyses

The *ca* and *ts* phenotypes of the viruses were assessed by evaluating viral replication in embryonated chicken eggs at permissive (32°C), restrictive (38°C) and low temperatures (25°C). Infectious titer of *wt* and MDVs at different temperature were measured by 50% egg infectious dose per milliliter (EID_50_/ml). Briefly, ten-fold serial dilutions of allantoic fluid were made in PBS (137 mM NaCl, 10 mM phosphate, 2.7 mM KCl, pH of 7.4) and 0.2 ml of each dilution was inoculated into 10-day-old SPF eggs. Five eggs were infected with each virus dilution and incubated at 32°C and 38°C for 48 h. and 6 days at 25°C. Harvested allantoic fluids were tested for virus presence by hemagglutination using 0.5% tRBC. The virus titer was calculated using Reed and Muench method [38]. Viruses that displayed ≥ 3 log_10_ reductions in viral titers at the restrictive temperature compared with that observed at the permissive temperature were considered ts. Viruses that displayed ≤ 3 log_10_ reductions in viral titers at low temperature were considered *ca*.

### Derivation of influenza viruses by reverse genetics

DNA from a set of eight plasmids was transfected into HEK293T cells using Lipofectamin2000 transfection reagent (Life Technologies) according to the manufacturer's protocol. Two days after transfection 0.4 μg/ml TPCK-treated trypsin (Sigma-Aldrich) was added to culture supernatants and after an additional 24 hours transfected cells were re-suspended in culture media and inoculated into embryonated chicken eggs. Allantoic fluid was harvested two days after inoculation and the presence of virus was revealed by agglutination of tRBC [36]. The serially diluted virus was inoculated into eggs to obtain C1E2 working stocks of reverse genetics-derived virus. The complete genome of virus derived by reverse genetics was sequenced. Reassortant viruses were generated in compliance with “NIH Guidelines for Research Involving Recombinant and Synthetic Nucleic Acid Molecules”.

### Genomic sequence analysis

The complete viral cDNAs for each segment (PB2, PB1, PA, HA, NP, NA, M and NS) of the cloned viruses were synthesized from purified viral RNA using SuperscriptTM III One-Step RT-PCR System with Platinum Taq High Fidelity (Life Technologies). Viral genome fragments (two for PB2, PB1, PA, NP, HA, and NA, and one for M and NS) were amplified using gene segment specific primers. RT-PCR products were resolved by the 2% E-Gel agarose electrophoresis system (Invitrogen, Carlsbad, CA) and were purified by ExoSAP-IT system (Affymetrix/USB, Cleveland, OH). Sanger sequencing of the cDNA was performed using the BigDye Terminator v3.1 Cycle Sequencing kit (AppliedBiosystems, Foster City, CA). The sequencing extension products were purified using the BigDye Xterminator Purification kit (Life Technologies) and analyzed using an ABI 3500xL DNA Analyzer (Applied Biosystems, Grand Island, NY, USA). Trace files were assembled in Sequencer (Gene Codes Corporation, Ann Arbor, MI). BioEdit Sequence Alignment Editor (Thomas Hall/Ibis Biosciences, Carlsbad, CA) software was used to align consensus sequences of gene segments with the corresponding reference sequences. The sequences of HA and NA were uploaded to Global Initiative on Sharing All Influenza Data (GISAID) EpiFlu database.

### Antigenic characterization

Antigenic characterization was performed using a panel of representative H7 wild type viruses/antisera as well as antisera generated against WHO candidate vaccine viruses. To generate ferret antisera against the LAIVs used for HI testing, serologically naïve, male ferrets greater than six months of age (Triple F Farms, Sayre, PA) were inoculated intranasally (i.n.) with 500 μl of diluted virus per nare (10^4^ or 10^6^ EID_50_ of either CDC-LV7A or A(H7N9)RG-LV1 virus, respectively). Ferrets were boosted in the hind limb with concentrated virus and adjuvant at 14 days post-infection (dpi) and were exsanguinated at 28 dpi. Ferret antisera against other H7 viruses tested by HI, including those previously identified as candidate vaccine viruses, were generated using the same strategy with a range of i.n. inoculation doses depending on the strain. Serum was stored at -20°C until further use. As previously described for the HI assay, viruses were standardized to 8 HAU/50ul and added to serially diluted, receptor destroying enzyme (RDE)-treated antisera (DENKA SEIKEN, Campbell, CA) followed by incubation at room temperature and agglutination with tRBC [37]. The HI titers were reported as the reciprocal of the last dilution of antiserum that completely inhibited hemagglutination.

### Pathogenicity testing in ferrets

All animal research was approved by the Centers for Disease Control and Prevention's Institutional Animal Care and Use Committee and conducted in an Association for Assessment and Accreditation of Laboratory Animal Care International-accredited animal facility. Six male adult Fitch Ferrets (Triple F Farms, Sayre, PA) that were serologically negative for currently circulating influenza viruses were used to determine the pathogenicity of the vaccine candidate. Ferrets were inoculated intranasally (1 ml) with virus diluted in PBS. For each virus, three animals were euthanized on day 3 post-inoculation (p.i.) to determine the extent of virus replication in multiple organs. Anesthesia occurred through an intramuscular injection of a ketamine-xylazine-atropine cocktail (25 mg/kg ketamine, 2 mg/kg xylazine, and 0.05 mg/kg of atropine). For euthanasia, ferrets were heavily sedated with the above cocktail, exsanguinated, and euthanatized via intracardiac injection of Euthanasia V solution (1 ml/kg). The heartbeat was monitored after delivery of Euthanasia V solution until the cessation of beating, at which time euthanasia was confirmed. The remaining 3 ferrets were monitored daily for clinical signs including body temperature, weight loss, respiratory symptoms, and lethargy for 14 days p.i. Clinical signs of infection did not exceed humane endpoints as measured by weight loss, lethargy, or appearance of neurologic symptoms. Nasal washes were collected from anesthetized ferrets on days 1, 3, 5, 7 and 9 (or 10) p.i., rectal swabs were collected on days 1, 3, 5 and 7 p.i. No analgesics were given during the course of infection as this would impact the ability to identify certain clinical signs of infection such as body temperature and lethargy or malaise. No ferrets experienced an unintended death or were humanely euthanized due to excessive illness observed during the course of the study.

## Results

### Generation of influenza A/H7N9 candidate vaccine viruses by classical reassortment

Live attenuated A(H7N9) influenza candidate vaccine virus was generated by classical reassortment in eggs by co-infection of *wt* A/Anhui/01/2013 (E2/E1) and MDV and genotyped using pyrosequencing. Based on the pyrosequencing analysis eggs 23 and 24 were selected for further cloning procedure (data not shown). The AF from eggs 23 and 24 were cloned by limiting dilution in the presence of antiserum to MDV, and twelve 6:2 reassortants were identified. The sequencing analysis of surface antigen genes of these clones revealed the variety of mutations in HA and NA genes. Clone 2383 with minimal number of changes in HA and NA genes (Table 1) was picked for further cloning (three times by limited dilution) and amplified to produce the A/Anhui/01/2013 (H7N9)-CDC-LV7A seed virus stock.

**Table 1:** Amino acid changes in HA and NA sequences of reassortants during generation of A/Anhui/01/2013-CDC-LV7A.

clones	2383	2386	2495	2485
**HA changes**	N123D* N149D	N123D N149D	N123D N149D L217Q	N123D N149DL217Q L447M
**NA changes**	T10I	T10I E411K	K460R	K460R

*H7 numbering.

Complete genome sequence analysis of CDC-LV7A followed by alignment of the six internal protein gene segments (PB2, PB1, PA, NP, M, and NS) with those of MDV A/Leningrad/134/17/57 and the two surface antigen segments (HA and NA) with the corresponding segments of the *wt* A/Anhui/01/2013 (E2/E1) was performed. The alignment of sequences for the six internal genes showed that all sequences were identical to those of MDV. The nucleotide sequence of HA gene of A/Anhui/1/2013 (H7N9) egg isolate E2/E1, which was used for reassortment, had a mixed population of N and D at position 123 (H7 numbering) compared to the HA sequence of *wt* A/Anhui/1/2013 from a human specimen (GISAID accession number EPI439507) which had N123. The HA gene of CDC-LV7A vaccine candidate had two egg adapted mutations, N123D and N1149D. The NA sequence of *wt* parental E2/E1 virus had a mixed population at position 10 (T or I) compared to original *wt* NA sequence (GISAID accession number 439509) which had 10T. The NA sequence of CDC-LV7A was identical to one of the *wt* parental E2/E1 virus with fixed T10I amino acid substitution. The sequences of the HA and NA genes of CDC-LV7A were deposited to GISAID EpiFlu Database—HA accession number EPI516488, NA accession number EPI516487.

### Generation of A(H7N9) candidate vaccine viruses by reverse genetics

The mutations present in the HA of a vaccine candidate seed virus could potentially affect its antigenicity and effectiveness [39, 40]. To evaluate the effect of selected-from-egg mutations on the antigenicity of the candidate vaccine virus we generated the recombinant viruses A(H7N9)RG-LV1 with HA and NA genes identical to *wt* Anhui/01/2013 E1 isolate and A(H7N9)RG-LV2 with mutations N123D and N129D in HA and T10I in NA (same as in CDC-LV7A) using the reverse genetics approach. The HA and NA genes of the A/Anhui/1/2013 virus were amplified by PCR from synthetic DNA amplicons, and cloned into a reverse genetics vector flanked by human polymerase I promoter and mouse RNA polymerase I terminator element [41, 42]. The mutations A367G and A445G in HA gene, which resulted in N123D and N149D changes in HA1 (H7 numbering) and C29T in NA gene that resulted in T10I change were introduced by site-directed mutagenesis. Generation of viruses was performed from 8 plasmid DNAs whereby 6 internal genes (PB2, PB1, PA, NP, M, NS) originated from MDV A/Leningrad/134/17/57 (H2N2) [43] and HA and NA originated from A/Anhui/1/2013. Infectious viruses were recovered from the transfected cells and passaged twice in eggs as described in Material and Methods. Full genome sequencing of the rescued viruses confirmed complete identity with reference sequences in all viral genes.

### Evaluation of egg adapted mutations on virus replication and phenotype

**Fig 1:**
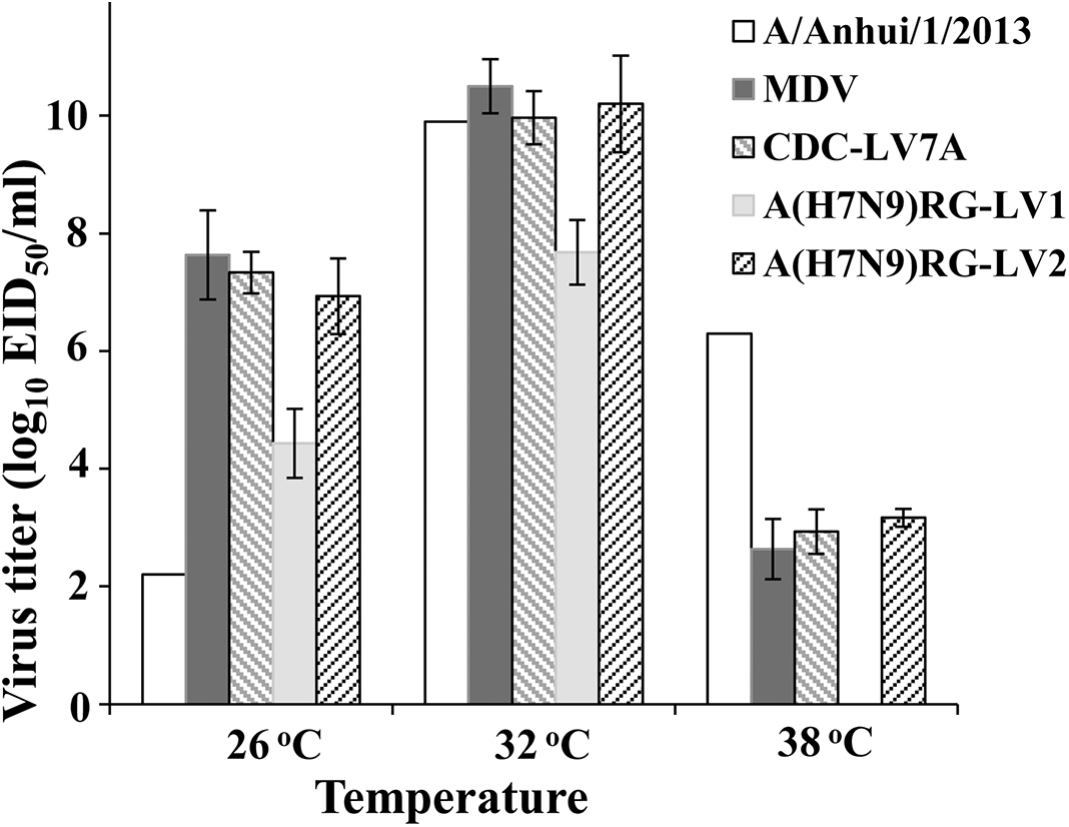
The growth characteristic of MDV, A/Anhui/1/2013 and 6:2 LAIV reassortants CDC-LV7A, A(H7N9)RG-LV1 and A(H7N9)RG-LV2 in eggs. The viruses were inoculated into eggs and grow at low (25°C), permissive (32°C), restrictive (38°C) temperatures. Infectious titers were measured by 50% egg infectious dose per milliliter (EID_50_/ml).

The important properties of live attenuated influenza vaccine include the limitation of replication of the vaccine viruses above 37°C and the effective replication at low temperatures (e.g., upper respiratory airway), thus providing temperature sensitive (*ts*) and cold adapted (*ca*) phenotype. In this study we analyzed the phenotypic characteristic of candidate vaccine virus CDC-LV7A and evaluated the selected-from-eggs mutations in HA and NA genes on their effect of virus replication. *Ts* and *ca* phenotypes of CDC-LV7A, A(H7N9)RG-LV1 and A(H7N9)RG-LV2 were analyzed by propagating viruses in SPF eggs at permissive (32°C), restrictive (38°C) and low (26°C) temperatures. Replicative efficiency of candidate vaccine virus CDC-LV7A and A(H7N9)RG-LV2 at the permissive temperature was similar to that detected for *wt* A/Anhui/1/2013 and MDV A/Leningrad/134/17/57 (10^10^, 10^10.2^ 10^9.9^ and 10^10.5^ EID_50_/ml respectively) (Fig 1). However replication efficiency of CDC-LV7A and A(H7N9)RG-LV2 at 26°C was significantly higher compare to that of *wt* virus (10^7.3^ and 10^6.9^ compare to 10^2.2^ EID_50_/ml) and was not different from MDV (10^7.7^ EID_50_/ml) confirming its *ca* phenotype. At restrictive temperature (38°C) replication efficiency of CDC- LV7A and A(H7N9)RG-LV2 was significantly reduced. It was seven log_10_ lower than at 32°C (10 ^2.9^ and 10^3.1^ EID_50_/ml compared to 10^10^ EID_50_/ml), five log_10_ lower than at 26C (10 ^2.9^ and 10^3.1^ EID_50_/ml compared to 10^7.3^ EID_50_/ml) and it was similar to replication efficiency of MDV (10^2.2^ EID_50_/ml). These results showed that recombinant H7N9-RG-LAIV2 virus was phenotypically indistinguishable from the CDC-LV7A reassortant—they both exhibited *ca* and *ts* phenotypes similar to MDV—i.e., replicated efficiently at low temperature (26°C), but not at 38°C. (Fig 1). Recombinant virus A(H7N9)RG-LV1 replicated less efficiently at 32°C and 26°C compared to A(H7N9)RG-LV2 and CDC-LV7A and did not replicate at 38°C. The results showed that the mutations present in surface protein genes of A(H7N9)RG-LV2 and CDC-LV7A (N123D (N129D in HA and T10I in NA) are responsible for better replication of the reassortants, since the absence of these mutations in A(H7N9)RG-LV1 reduced its replication efficiency in eggs by three log_10_ at 26°C (10 ^4.4^ compare to 10^7.7^and 10 ^6.9^ EID50/ml) and 38°C (10 ^3.1^ compare to 0 EID_50_/ml) and more than two log_10_ at 32°C (10^7.7^ compare to 10^10^ EID_50_/ml).

### Genetic homogeneity testing of CDC-LV7A

The genetic homogeneity of CDC-LV7A was demonstrated by real-time RT-PCR as described previously [35]. The specificity tests demonstrated no cross-templates reactivity, confirming that the designed primers/probe sets were strain specific. The sensitivity of the assays was determined and was shown to be as low as 1 EID_50_ per reaction. Homogeneity of CDC-LV7A seed candidate vaccine virus was analyzed using RNA corresponding to10^5^ EID_50_/reaction. MDV and *wt* viruses were also tested as controls. The positive signals were detected only with primers/probe specific for internal genes of MDV and HA and NA genes for A/Anhui/01/2013 (Table 2). Homogeneity of candidate vaccine virus was also characterized after five and ten serial passages in eggs. The RNAs isolated from CDC-LV7A-E5 and CDC-LV7A-E10 was subjected to real time RT-PCR. The reactions with RNA from passaged reassortants gave positive signals only with primers/probe specific for internal genes of MDV and HA and NA for *wt* with C_t_ values similar to the ones obtained for non-passaged virus (Table 2). No signal was detected in the reactions with RNA from passaged reassortant when primers/probe sets for internal genes of *wt* and HA and NA for MDV were used, confirming that the reassortant virus CDC-LV7A obtained by classical reassortment is genetically homogeneous.

**Table 2:** Cycle threshold (C_t_) values (±Std. Dev) of CDC-LV7A RNA corresponding to 10^5^ EID_50_/per reaction.

Gene	Assays specific for A/Leningrad/134/17/57		Assays specific for *wt* A/Anhui/1/2013	
PB2	23.3±0.1	25.2±0.1	No C_t_	No C_t_
PB1	25.9±0.3	27.1±0.2	No C_t_	No C_t_
PA	24.1±0.1	25.1±0.2	No C_t_	No C_t_
NP	23.3±0.5	24.4± 0.1	No C_t_	No C_t_
M	31.1±0.7	28.1±0.1	No C_t_	No C_t_
NS	24.9±0.1	26.0± 0.1	No C_t_	No C_t_
HA	No C_t_	No C_t_	25.6±0.2	26.4±0.3
NA	No C_t_	No C_t_	27.9±0.3	27.9±0.2

### Genetic stability of A(H7N9) CDC-LV7A reassortant

Genetic stability of CDC-LV7A was analyzed in two host models: chicken eggs and ferrets. During vaccine manufacturing, the number of passages the LAIV candidate virus undergoes, should not exceed 4–5 [44], however CDC-LV7A candidate vaccine virus was subjected to ten passages in embryonated chicken eggs in two independent experiments. RNA was isolated from the viruses after five and ten passages (CDC-LV7A-E5 and CDC-LV7A-E10 respectively). CDC-LV7A-E5 and CDC-LV7A-E10 were characterized by complete genome sequencing. Sequence alignments of the eight gene segments of the CDC-LV7A-E5 viruses with the original CDC-LV7A did not reveal any nucleotide differences between the sequences in both independent trials, indicating the stability of the reassortant genome. However, alignments of the eight gene segments of the CDC-LV7A-E10 viruses with the original CDC-LV7A revealed the presence of quasispecies population in HA1 at position 196 –G/E (original reassortant had G196, H7 numbering), at position 217 –L/Q (original reassortant had L217) and one fixed mutation L447M in CDC-LV7A-E10-1 virus, while CDC-LV7A-E10-2 isolate did not have any mutations even after ten passages. The rest of both CDC-LV7A-E10 genes viruses did not have any substitutions (Table 3).

**Table 3:** Amino acid changes in A/Anhui/01/2013(H7N9)-CDC-LV7A before and after passaging in eggs.

Segment	Passage 0 and 5	Passage 10
**HA**	N123D, N149D*	N123D, N149DG196G/E, L217L/Q, L447M
**NA**	T10I	T10I
**PB2**	Identical to MDV	Identical to MDV
**PB1**	Identical to MDV	Identical to MDV
**PA**	Identical to MDV	Identical to MDV
**NP**	Identical to MDV	Identical to MDV
**M**	Identical to MDV	Identical to MDV
**NS**	Identical to MDV	Identical to MDV

*H7 numbering.

The genetic stability of CDC-LV7A was also analyzed in ferrets. Three animals were vaccinated intranasally; the virus samples taken from nasal washes on day 5 and 7 were analyzed. Full genome sequence was performed for all three viruses on day 5 and no changes were detected in any virus genes. The virus titers in nasal washes on day 7 were extremely low and amount of RNAs purified from these samples were sufficient only for sequencing of HA and NA genes but not internal genes. No changes were detected in HA or NA gene sequences in all three animals on day 5 and 7. These analyses provide additional support of genetic stability of CDC-LV7A candidate vaccine virus.

### Evaluation of antigenicity of the H7N9 reassortant viruses in ferrets

Polyclonal ferret antisera was raised against CDC-LV7A and A(H7N9)RG-LV1 to assess their antigenicity. Two-way HI cross-reactivity of reassortant viruses were compared to one another, to the *wt* A/Anhui/1/2013 (H7N9), A/Netherlands/219/2003 (H7N7), A/Mallard/Netherlands/12/2000 (H7N3) and A(H7N9) reference viruses isolated from 2013 to 2015. The HI titers of antisera generated against reassortant viruses were within a two-fold difference to the wild type reference viruses, suggesting that both reassortants were antigenically related to the wild type A/Anhui/1/2013 and could be considered as candidate vaccine viruses (Table 4). Antigenic relationships between candidate vaccine viruses and heterologous H7N9 viruses isolated in 2013–2015 were also evaluated. Ferret antiserum raised against both viruses inhibited the majority of recently circulating viruses, with HI titers equal to or within a two-fold difference for A(H7N9)RG-LV1 antisera and up to four-fold differences for CDC-LV7A antisera. The antisera to reference virus A/Anhui/1/2013 and both vaccine candidates cross-reacted with A/Netherlands/219/2003 (H7N7) virus with a titer reduction of ≥ 8-fold (Table 4), indicating that reassortants and current A(H7N9) viruses are antigenically distinct from this Eurasian lineage influenza A(H7N7) viruses. However, a 4-fold reduction in HI titers was observed when both serum were tested against av-A(H7N3) A/Mallard/Netherlands/12/2000 virus, suggesting that reassortants and current A(H7N9) viruses are antigenically related to this Eurasian lineage influenza A(H7N3) viruses.

**Table 4:** Two-way hemagglutination inhibition assay of reference A(H7N9) viruses and LAIV reassortants.

		REFERENCE FERRET ANTISERUM								
		NL/219/03^	ML/NL/12	Anhui/1/13	SH/2/13	TW/1/13	HK/5942/13	CDC-LVRG1	CDC-LV7A	Passage^a^
**REFERENCE STRAINS**										
H7N7	A/Netherlands/219/2003	**160** ^**b**^	5	20	80	80	10	40	80	EX/E2
av-H7N3	A/Mallard/Netherlands/12/2000	640	**160**	160	160	640	160	160	320	E2/E2
H7N9	A/Anhui/1/2013	320	160	**320**	640	1280	160	640	640	E2/E1
H7N9	A/Shanghai/2/2013	160	160	160	**160**	320	80	640	320	E1/E1
H7N9	A/Taiwan/1/2013	320	320	320	640	**1280**	80	1280	640	E1/E1
H7N9	A/Hong Kong/5942/2013	320	160	160	320	640	**320**	320	640	M3/E1
**REASSORTANTS**										
H7N9	A/Anhui/1/2013- CDC-LVRG1	320	160	160	640	640	160	**640**	320	C1/E2
H7N9	A/Anhui/01/2013-CDC-LV7A	320	320	320	640	1280	160	640	**1280**	E2/E1// SPF7
**TEST ANTIGENS**										
H7N9	A/Shanghai/1/2013	320	80	160	320	640	80	640	320	E1/E1
H7N9	A/Jiangsu/1/13	320	160	320	640	640	160	1280	640	E2/E1
H7N9	A/Zhejiang/2/2013	320	320	320	1280	2560	320	1280	320	E1/E2
H7N9	A/Shanghai/7/2013	320	160	320	640	1280	320	1280	640	EX/E1
H7N9	A/Hong Kong/2212982/2014	640	320	320	1280	2560	320	1280	640	M1/E1
H7N9	A/Hong Kong/734/2014	320	160	320	1280	2560	320	1280	640	M1/E1
H7N9	A/Hong Kong/56/2015	320	80	320	320	1280	160	640	320	OR/E1
H7N9	A/Hong Kong/2550/2015	160	80	160	320	1280	160	1280	320	C1/E1
H7N9	A/British Columbia/2015	640	320	320	1280	1280	320	1280	1280	C1/C1

^a^ E—eggs, SPF–special pathogen free eggs, C–cells, M–MDCK cells, OR–original specimen

^b ^Homologous titers of ferret antiserum to reference antigen are indicated in bold

^ The complete names of the antigen are listed under the reference strains.

### Characterization of pathogenicity and replication in ferrets

Vaccine safety was studied in the ferret model. Three animals were infected intranasally with 10^6^ plaque forming units (PFU) of *wt* A/Anhui/1/2013 or 10^6.1^ PFU of A/Anhui/1/2013 (H7N9)-CDC-LV7A virus. Morbidity was reduced in ferrets infected by the A/Anhui/1/2013 (H7N9)-CDC-LV7A vaccine candidate compared to the *wt* A/Anhui/1/2013 virus. Peak virus shedding in nasal washes was observed on day 1 p.i. for both groups of ferrets but was 83-fold lower in animals infected by the candidate vaccine virus (Table 5). *Wt* A/Anhui/1/2013 virus replicated efficiently throughout the respiratory tract of ferrets while A/Anhui/1/2013 (H7N9)-CDC-LV7A virus was limited to the upper respiratory tract of 3/3 animals. No virus was detected in any extrapulmonary tissues tested from animals infected by the A/Anhui/1/2013 (H7N9)-CDC-LV7A candidate vaccine virus (Table 5). The results confirmed that A/Anhui/1/2013 (H7N9)-CDC-LV7A was attenuated compared to *wt* A/Anhui/1/2013.

**Table 5:** Clinical signs and virus replication of A(H7N9) *wt* and LAIV viruses in ferrets.

	Clinical signs				Virus replication (titers)^§^			
Virus	Temp rise (C°)*	Weight loss^¶^ (%)	Anorexia^#^	Lethargy^#^ (day 2 pi)	Nasal wash (day 1 pi)	Nasal turbinates (day 3 pi)	Lungs (day 3)	Rectal swabs (day 5)
A/Anhui/1/2013	0.7–2.9	10.9	3/3	3/3	7.0 (3/3)	6.3 (3/3)	5.7 (3/3)	2.4 (3/3)
CDC-LV7A	0.4–0.6	3.3	0/3	0/3	5.08 (3/3)	5.08 (3/3)	0	0

*Temperature increase (range) over baseline during the first 10 days pi.

^**¶**^ The percentage mean maximum weight loss observed during the first 10 days post infection (pi)

^**§**^ Peak mean log_10_(p.f.u.ml^-1^) for *wt* or log_10_(EID_50_ml^-1^) for LAIV

^**#**^Number of ferrets showing anorexia and lethargy is shown.

## Discussion

The ongoing A(H7N9) influenza virus outbreak in China has renewed concerns about zoonotic influenza viruses with pandemic potential. WHO regularly updates antigenic and genetic characteristics of circulating zoonotic influenza viruses and recommends development of candidate vaccine viruses (CVVs) for pandemic preparedness. CVVs available for distribution to manufacturers of inactivated influenza vaccine are listed and updated twice annually (http://www.who.int/influenza/vaccines/virus/characteristics_virus_vaccines/en/). To date all A(H7N9) reassortants available for distribution by WHO were generated by reverse genetics.

The current report describes the generation of A(H7N9) LAIV obtained by classical reassortment in eggs. The vaccine candidate A/Anhui/1/2013(H7N9)-CDC-LV7A virus was shown to grow to high titers in eggs and was antigenically similar to *wt* A/Anhui/1/2013(H7N9) virus. Since this vaccine seed virus was prepared by classical reassortment, it was important to confirm the absence of any genetic material of undesired origin and to demonstrate that there were no contaminating *wt* internal genes present that may allow efficient lower respiratory replication in vaccinees that may cause illness. Likewise, it was critical to demonstrate that HA or NA genes from the MDV were not present which might influence vaccine effectiveness. CDC-LV7A seed virus was shown to be homogeneous by real time RT-PCR analysis. To ensure the quality of the vaccine seed virus and to demonstrate the stability of the vaccine reassortant genome composition and sequence, sequencing of the reassortants was performed after five and ten passages in eggs. No mutations in any genes were detected after five passages. However, after ten passages a number of new egg adapted mutations was detected. This suggested that passaging of the vaccine seed virus during the manufacturing process should not exceed five passages since the HA gene might acquire additional egg adapted changes.

Since eggs are the only currently approved substrate for vaccine production, reassortants generated by reverse genetics must still be passaged several times in eggs, hence acquiring egg adapted mutations. These mutations, which usually improve vaccine virus yield, might affect vaccine immunogenicity. Thus, continuous analysis for mutations in viruses used for vaccine production is critical. During the vaccine manufacturing process the seed virus undergoes four to five additional passages before blending the pool to formulate LAIV [44]. The lack of the mutations in sequences of CDC-LV7A virus passaged five times indicates the high level of its genetic stability. However, in one of the stability experiment, a number of new egg adapted mutations was detected after ten passages. This suggested that LAIV vaccine manufacturers should strictly adhere to the current regulations not to exceed five passages of the seed viruses. The reassortant virus A(H7N9)-RG-LV1 containing the *wt* A/Anhui/1/2013 HA and NA gene sequences replicated poorly in eggs compared to the classically generated reassortant CDC-LV7A (or A(H7N9)-RG-LV2)—i.e., only up to 10^7.7^ EID_50_/ml at 32°C; these levels are analogous to a H7N9 LAIV candidate based on A/Ann/Arbor/6/60 [24] and PR8-based H7N9 reassortant RG32A [24]. An H7N9/PR8 split candidate vaccine virus developed in China grew in eggs also to a comparable level of 10^8.5^ EID_50_/ml [20]. LAIV candidate CDC-LV7A containing the HA N123D and N149D mutations grew to a much higher titers in eggs (10^10^ EID_50_/ml), which is very significant for manufacturing, when from the same amount of eggs allantoic fluid almost 100 times more doses of live vaccine could be produced.

Critical vaccine properties, however, are antigenic relatedness to circulating viruses and protective immunogenicity. Therefore, vaccine seed viruses must pass a two-way HI test to be considered for vaccine production [45, 46]. In a two-way HI test, the reassortant virus is considered consistent with wild type reference virus if ferret antisera generated against the vaccine strain reacts with the *wt*, parental virus at titers equivalent to or within a two-fold reduction to the homologous virus titer and vice versa. Post-infection ferret antisera were raised against CDC-LV7A and A(H7N9)RG-LV1 viruses to assess their antigenicity. Two-way HI testing showed that sera against both reassortants efficiently inhibited hemagglutination by the homologous A(H7N9) virus as well as the parental virus indicating that mutations in HA and NA of CDC-LV7A did not affect antigenicity of the vaccine candidate. Moreover, the antisera against both reassortants also inhibited hemagglutination (within four-fold of homologous titers) by all *wt* A(H7N9) isolates tested including viruses collected in 2013, 2014 and 2015, indicating that CDC-LV7A seed virus is an ideal LAIV candidate vaccine.

The high LAIV yield in eggs and ‘needle-free’ nasal administration makes a LAIV a desirable vaccine for use. Since 2009, WHO was licensed to develop, register, manufacture, use and sell seasonal and pandemic LAIV based on Russian MDVs. It transferred the technology to produce LAIV by classical reassortment in eggs to manufacturers in India, Thailand and China [10–12]. The agreement allows for all CVVs generated on the backbone of the Russian MDVs by IEM, Russia and/or CDC, USA to be available in these countries. In regards to A(H7N9) LAIV CVV described here, availability is currently most important in China, which continues to be affected by annual waves of A(H7N9) human cases.

## Supporting Information

S1 Table Primers and probes used for real-time RT-PCR amplification of A/Anhui/01/2013 (H7N9) genes. (DOCX)
