# Supplementary material for: Generation and Characterization of Live Attenuated Influenza A(H7N9) Candidate Vaccine Virus Based on Russian Donor of Attenuation
Source: PLoS One. 2015 Sep 25;10(9):e0138951. doi: 10.1371/journal.pone.0138951 (PMC4583547; doi:10.1371/journal.pone.0138951)
Supplement: S1 Table — (DOCX) [file pone.0138951.s001.docx]

S1 Table. Primers and probes used for real-time RT-PCR amplification of A/Anhui/01/2013 (H7N9) genes.

| Primers/probes* | Gene | | Nucleotides | | Sequence 5´ -> 3´ |  |
| --- | --- | --- | --- | --- | --- | --- |
|  |  |  | |  | | |
| HAAnh1F915 | HA | 915-937 | | CATAGATAGCAGGGCAGTTGGAA | | |
| HAAnh1R981 | HA | 960-981 | | TGTTGCTAGCAGCAGACTCCTT | | |
| HAAnh1P939 | HA | 939-958 | | FAM-ATGTCCGAGATATGTTAAGC -BHQ1 | | |
| NAAnh1F405 | NA | 405-427 | | AATCAGAGGGAAACACTCAAACG | | |
| NAAnh1R468 | NA | 449-468 | | GCTTATCAGGGCGCGATACT | | |
| NAAnh1P429 | NA | 429-447 | | FAM-AACAATACACGATAGGTCC -BHQ1 | | |
| MAnh1F573 | M | 573-594 | | GATGGCTGGATCAAGTGAACAG | | |
| MAnh1R633 | M | 614-633 | | TTGCCTAGCCTGACTTGCAA | | |
| MAnh1P596 | M | 596-609 | | FAM-CAGCGGAAGCCATG -BHQ1 | | |
| NSAnhiF143 | NS | 143164 | | GCACTCTTGGTCTGGACATCAG | | |
| NSAnh1R206 | NS | 183-206 | | AAAATCCGCTCCACTATATGCTTT | | |
| NSAnh1P168 | NS | 168-181 | | FAM-TGCCACGCGTGAAG -BHQ1 | | |
| NPAnh1F832 | NP | 832-852 | | GCTTGTGTGTACGGACTTGCA 832 | | |
| NPAnh1R895 | NP | 872-895 | | CCAAGGAGTACCCTTCTCTCTCAA | | |
| NPAnh1P854 | NP | 854-870 | | FAM-TGGGTACGACTTCGAAAA-BHQ1 | | |
| PAAnh1F1651 | PA | 16511671 | | CGAACTGCAGTAGGCCAAGTG | | |
| PAAnh1R1714 | PA | 1695-1714 | | TGGAGGTCCCATTGGTTCTC | | |
| PAAnh1P1690r | PA | 1673-1690 | | FAM- CAAGACCCATGTTTCTGT -BHQ1 | | |
| PB1Anh1F413 | PB1 | 413-429 | | CGGCTGCTACCGCATTG | | |
| PB1Anh1R471 | PB1 | 453-471 | | GGCTGTCAGGCCATTCGAT | | |
| PB1Anh1P431 | PB1 | 431-451 | | FAM- CCAACACTATAGAGGTATTCA -BHQ1 | | |
| PB2 Anh1F391 | PB2 | 391-409 | | GGCCCCGTTCACTTCAGAA | | |
| PB2Anh1R454 | PB2 | 436-454 | | CATGGCCCGGGTTTATGTC | | |
| PB2Anh1P412 | PB2 | 412-432 | | FAM- CAGGTTAAAATACGCCGCAGG -BHQ1 | | |

* F and R in the primer name indicate forward and reverse direction, respectively. P indicates probe, and r indicates probe in reverse direction.
